# Assessment of reproductive, genotoxic, and cytotoxic effects of leachate-contaminated water in male mice

**DOI:** 10.1016/j.heliyon.2024.e40126

**Published:** 2024-11-05

**Authors:** Ranjit Kakati, Kamal Adhikari, Queen Saikia, Ajit Hazarika

**Affiliations:** a Department of Zoology, Gauhati University, Guwahati, India; b Department of Zoology, Tihu College, Tihu, Nalbari, Assam, India; c Department of Zoology, Mangaldai College, Mangaldai, Darrang, Assam, India; d Tyagbir Hem Baruah College, Jamugurihat, Sonitpur, Assam, India

**Keywords:** Waste dumping, Leachate, Reproductive toxicity, Oxidative stress

## Abstract

Leachate-contaminated water (LCS) poses significant health risks due to its heavy metal content and altered physicochemical properties. This study examined the physicochemical parameters and heavy metal levels in LCS and assessed its reproductive toxicity, genotoxicity, and cytotoxic effects in exposed mice. Groups of mice (n = 5) were orally administered 100 μL of 30 % and 70 % LCS (v/v) twice daily for 35 days. Drinking water served as a negative control, and cyclophosphamide (Cyp) (20 mg/kg bw) as a positive control. On day 36, the mice were weighed, sacrificed, and their testicular weight, sperm count, sperm morphology, viability, acrosome integrity, and serum testosterone were examined. Oxidative stress in the testes, histopathological changes, and serum markers for liver and kidney function (SGOT, SGPT, and creatinine) were also assessed. Genotoxic effects were evaluated using a micronuclei (MN) assay. Analysis of the leachate showed altered physicochemical parameters and elevated heavy metal levels. Exposure to LCS led to a significant decrease in relative testis weight, sperm count, normal sperm morphology, viability, acrosome integrity, and serum testosterone levels. It also caused a notable increase in MDA levels and a decrease in catalase (CAT), superoxide dismutase (SOD), and glutathione (GSH) levels, along with histological changes in the testes of LCS-treated mice compared to controls. Additionally, there was a significant rise in MN formation in RBCs and elevated levels of liver enzymes and creatinine, indicating liver and renal toxicity. Histological alterations in the liver and kidneys were also observed in LCS-exposed mice. These findings suggest that LCS induces reproductive toxicity, genotoxicity, and cytotoxicity in male subjects.

## 1 Introduction

The global population is increasing at an alarming rate, and with this surge, the amount of waste generated per capita is also rising proportionally. This phenomenon presents significant challenges for waste management systems worldwide and has far-reaching implications for the environment, public health, and sustainable development. The substantial amount of waste generated daily from residential and commercial areas, industries, and agriculture falls under a specific category known as municipal solid waste (MSW) [1]. Currently, approximately 2 billion tons of MSW are produced worldwide, with 33 % of it still needing to be collected. In India, only 21 % of MSW is effectively managed, as landfills require significant land resources each year [1]. The remaining MSW is disposed of in open land without proper reprocessing [2]. This practice leads to the emission of foul odors, methane, and the production of leachate, which can contaminate the surrounding water [3]. Leachate is a highly concentrated liquid generated by these landfills, containing a mixture of organic, inorganic, and heavy metals [[4], [5], [6]]. Heavy metals pose a serious threat to human health and the aquatic ecosystem due to their toxicity, non-biodegradability, potential for bioaccumulation in living organisms, and carcinogenic properties. When released into the environment, heavy metals accumulate in various species, including humans, animals, and plants, causing harm [7].

Guwahati, a city with a population of about 1.26 million generates around 500 tons of garbage daily [8]. The MSW produced in Guwahati is disposed of at West Boragaon (26° 08′ 14.30″ N and 91° 38′ 51.80″ E) [9]. This disposal site is an open, mixed-use landfill near Deepor Beel, a Ramsar site (The Ramsar list, 2020; Ramsar site No. 1207). Unfortunately, the leachate containing various heavy metals generated in the dumping area is regularly released into the waters of Deepor Beel [9,10]. Leachate contamination often have adverse effects on the reproductive parameters of males [4,11], resulting in conditions such as tetrazoospermia and double-headed sperm [12]. The leachate also exert systemic toxicity and genotoxicity [13,14]. Approximately 14 villages, encompassing 1200 local families, depend on this wetland for their livelihoods, engaging in activities such as farming, fishing, and the gathering of medicinal plants and flowers [15]. Our survey revealed that these villagers grapple with various health problems, including issues related to systemic toxicity and infertility. To date, no study has explored the impact of MSW leachate from Deepor Beel on the reproductive health of mammals. Therefore, we have chosen the Deepor Beel as our study site to assess the effect of leachate contamination on the reproductive parameters of male mice. Our objective in this study is to assess the impact of LCS on the histopathology of the mouse testis and epididymal sperm parameters. We also want to know if LCS can alter the stress oxidative markers. Additionally, liver and renal histopathology will be addressed.

## 2 Materials and methods

### 2.1 Water sample analysis

LCS from nine different sites were collected at the Boragaon Garbage dumpyard, which connects to the Deepor Beel wetland. From each collection site, 100 ml of LCS was collected in a sterile sample bottle. Samples from all the collection sites were filtered through Whatman No. 42 filter paper to remove suspended particles, mixed in a 1 L sterile sample bottle to obtain a homogeneous LCS solution, and stored in the laboratory for subsequent experiments. Daily 10 ml of LCS was separated and used as an aliquot for animal experiments. It was centrifuged at 8000 rpm for 10 min, and the resulting supernatant was used as the stock solution.

We determined various physicochemical parameters using specific equipment. Electronic conductivity, Total Dissolved Solids, Turbidity, and pH values were measured with a Multiparameter Water Quality Analyzer (Model: Systronics-371). Sodium (Na^+^) and potassium (K^+^) concentrations were determined in the laboratory using a Flame Photometer (Model: Systronics-128) [16,17]. Biochemical oxygen demand was estimated in Oxitop (Model: WTW, i IS 6) and fluoride concentration was calculated using an ion meter (Model: Orionstar A214), dissolved oxygen (DO) was measured instantly using the Winkler method [16], and nitrate (NO_3_^−^) was quantified using a UV–Visible spectrophotometer (Model: Genesys 10 S, Thermofisher).

Before conducting a heavy metals analysis, the samples were acidified with concentrated nitric acid (HNO_3_) to lower its pH to below 2.0 and chilled to below 4 °C [18]. The total amount of all heavy metals was determined according to standard protocols using atomic absorption spectrometer (Model: iCE 3500, Thermofisher) [16,19].

### 2.2 Chemicals

SGOT (Serum Glutamic Oxaloacetic Transaminase) SGPT (Serum Glutamic Pyruvic Transaminase), and Creatinine kits was purchased from Aspen Laboratory Pvt. Ltd (Delhi, India). Testosterone kit (Catalog No. SE120119) was purchased from Sigma-Aldrich. All other chemicals required for the experiment were obtained from the chemical store of the Department of Zoology, Gauhati University.

### 2.3 Experimental subjects: Male mice

For the experimental purpose, adult, fertile, healthy mice 11- to 13-week-old, weighing 27 ± 3 g, were obtained from the Animal House Facility, Gauhati University. The Institutional Animal Ethics Committee (IAEC), Gauhati University, permitted the rational use of mice (Permit No. IAEC/ZOO/2022 PR-IAEC/RK/2022-7/01).

Before commencing the experiment, all the animals were acclimated in a separate hygienic room within the Animal House for 15 days. Five mice were kept per pathogen-free sterile plastic cages, maintained at a controlled temperature (27 ± 2 °C), with a 12/12-h light/dark cycle. They were given a routine diet (twice daily) and had access to water ad libitum. The experiment followed the guidelines of the Committee for the Purpose of Control and Supervision of Experiments on Animals (CPCSEA).

### 2.4 Selection of dose

The dose of LCS was determined based on a pilot study. In the pilot study, mice were divided into five groups (n = 5). Each group received 100 μL of LCS orally at different concentrations: Group I (20 % LCS), Group II (40 % LCS), Group III (60 % LCS), Group IV (80 % LCS), and Group V (100 % LCS) twice daily. A negative control group was treated with purified normal drinking water. These experiments were conducted for 35 days, followed by a fertility test.

For the fertility test, each male mouse was paired with two estrus-induced female mice in separate breeding cages, and the appearance of a vaginal plug was considered day zero of gestation. The mice were kept in separate cages until the birth of pups. Fertility rates were determined using the formula described by Ajayi and Akhigbe [20].

The pilot study revealed that the highest dose, 100 % LCS, was detrimental and sometimes lethal to the subjects. 80 % dose was detrimental but not lethal, while 60 % and 40 % LCS caused a significant decrease in fertility. Again, 20 % dose resulted in an insignificant decrease in fertility.

We selected a 35-day duration for the study, as the total spermatogenic cycle requires approximately 34.5 days.

Based on the pilot study's findings, we chose to investigate the effects of 30 % and 70 % LCS for the same 35-day duration.

### 2.5 Grouping of experimental animals

Male mice were divided into four groups (n = 5) and received oral LCS treatment as detailed in Table 1. All male mice were orally administered 100 μL of 30 % and 70 % LCS twice daily – once in the morning (8:00–9:00 Hrs) and once in the evening (16:00–17:00 Hrs). The negative control group received water only, while the positive control group was given 20 mg/kg body weight (bw) of Cyp orally for 35 days [21], both for 35 days. A literature survey showed that 20 mg/kg bw of Cyp is the minimum dose to induce reproductive toxicity and cytotoxicity [21,22], so this dose was used. On day 36, all animals were weighed, euthanized with ketamine hydrochloride, and blood serum and tissues were collected for further experiments.

**Table 1:** Different treatment groups of male mice.

Group	Group size	Dose	Duration (Days)	Dose (twice daily)
Negative Control	5	0 %	35	100 μL
Positive control (Cyp)		20 mg/kg bw		
T1 (LCS)		30 %		
T2 (LCS)		70 %		

### 2.6 Collection of blood serum

Blood sample was collected from the mice via cardiac puncture and placed in sample bottles without anticoagulant. The samples were centrifuged at 6000 rpm for 10 min, and the supernatant was used to measure testosterone, SGOT, SGPT, and creatinine levels.

### 2.7 Collection of organs

The testes were dissected for histological and antioxidant evaluation. The kidneys and liver were processed for histological analysis, while the epididymis was used for sperm parameter analysis.

### 2.8 Determination of testicular weight

The relative weight of the testis was measured using a high-precision electronic balance (Model: PGB200, Wensar).

### 2.9 Sperm parameters

Sperm parameters, including sperm count, abnormalities, acrosome status, and sperm viability, were evaluated using standard methods at 37 °C.

#### 2.9.1 Sperm suspension

The caudal epididymis was separated, cleaned, and minced in 1 mL of normal saline. The sperm from the caudal epididymis was then diluted in a 1:20 ratio (sperm to saline) to create a suspension [23].

#### 2.9.2 Sperm count

The sperm count was performed using the method described by Narayana et al. [24]. A 200 μL sample of the sperm suspension was placed in a Neubauer chamber, and sperm were counted under a light microscope at 40 × magnification across five squares. The sperm count was expressed as the number of spermatozoa per mL.

$$\begin{array}{c} \text{Sperm}/\text{mL}=\text{Number}\;\text{of}\;\text{sperm}\;\text{counted}\;\left(y\right)\times \text{dilution}\;\text{factor}/\text{volume}\times 1000\\ \text{Sperm}/\text{mL}=y\times 20/0.02\;\text{mm}3\times 1000\;\text{mm}3/\text{mL}\;=y\times 1000,000\;\text{Sperm}/\text{mL} \end{array}$$

#### 2.9.3 Sperm morphology

To assess sperm head and tail abnormalities, the method by Narayana et al. [25] was followed. A drop of sperm suspension was smeared on a clean slide, fixed with methanol for 2–3 min, and stained with 1 % Eosin to distinguish normal from abnormal sperm. A total of 1000 sperm per animal were evaluated according to the standards set by Wyrobek and Bruce [26]. The examination was conducted using a Leica DM 750 light microscope at 40× magnification. The mean percentage of abnormal sperm was calculated using the formula -

$$\text{Percentage}\;\text{of}\;\text{abnormal}\;\text{sperm}=\frac{Number\;of\;sperm\;with\;aberrations}{Total\;number\;of\;sperm\;studied}\times 100$$

#### 2.9.4 Sperm viability

Sperm viability was assessed using the method by Golshan-Iranpour et al. [27]. A 1:1 mixture of 5 μL 1 % eosin Y stain and 5 μL sperm suspension was placed on a slide and examined under a light microscope (Leica DM-750) at 40 × magnification. Motile (living) sperm remained unstained, while non-motile (dead) sperm absorbed the dye. Stained and unstained sperm were counted, and the ratio of living to dead spermatozoa was calculated to estimate viability:

$$\text{Percentage}\;\text{Viability}=\frac{Number\;of\;live\;spermatozoa}{Number\;of\;spermatozoa\;observed\;uder\;microscope}\times 100$$

#### 2.9.5 Acrosome assessment

We followed the method outlined by Nixon et al. [28] to assess acrosome status. Spermatozoa from the epididymis were fixed in a 4 % paraformaldehyde solution at a pH of 7.4 for 10 min. After fixation, the sperm were washed twice with 100 mM ammonium acetate (pH 9.0) and resuspended in 1 mL (100 mM) ammonium acetate. A 50 μL suspension of this was smeared on a glass slide. The smear was allowed to dry and then stained with 0.22 % Coomassie brilliant blue. Once again, the slide was air-dried, and a thin cover slip was placed over a drop of glycerol. The prepared slide was then observed under the microscope.

### 2.10 Assessment of antioxidants and oxidative stress marker

#### 2.10.1 CAT, SOD activity and GSH concentration

We measured the antioxidant levels using spectrophotometer (Model: Double beam Spectrophotometer 2203, Systronics).

We followed the method outlined by Bonaventura et al. [29] to evaluate CAT activity. In the testicular homogenate, we added hydrogen peroxide (H_2_O_2_) and measured the change in absorbance spectrophotometrically at 240 nm.

We followed the method outlined by Misra and Fridovich [30] to estimate SOD activity, recording the absorbance at 560 nm.

Using the procedure outlined by Ellman [31] with a few modifications, the GSH concentration in the testicular homogenates was determined. All the changes in the absorbance were measured using a spectrophotometer.

#### 2.10.2 Lipid peroxidation

Malondialdehyde (MDA), an end product of lipid peroxidation, is a biomarker of oxidative stress. TBARS, a red substance produced in the reaction between MDA and thiobarbituric acid, was detected spectrophotometrically at 532 nm following the method by Buege and Aust [32].

### 2.11 Testosterone assay

The testosterone kit was purchased from Sigma-Aldrich, and the protocol provided in the kit was followed to estimate the amount of testosterone in the serum. The ELISA plate was coated with anti-mouse IgG conjugated with testosterone-horseradish-peroxidase. The mouse serum was poured over the well and allowed to incubate. Unbound horseradish-peroxidase-testosterone and testosterone were washed away using a wash buffer. After washing, only the bound complex remained fixed in the wells. Tetramethylbenzidine substrate (a dye) was added to all the wells. Finally, a stop solution was added, and within 15 min, absorbance was measured at 450 nm in an ELISA reader (Model: BGS-291, Genetix). The limit of detection of the kit was 0.1 ng/ml.

### 2.12 Histological analysis of testis

Histological observations were performed as described by Saikia et al. [23]. After fixing in 10 % neutral buffer formalin overnight, the testicular tissues were rinsed in tap water for about 2 h. The tissues were then passed through ascending alcohol grades, followed by xylene and three further paraffin changes. The tissues were incubated in paraffin overnight. Following these, the tissues were embedded in paraffin blocks. The blocks were cut into 4 μm thick sections using a rotary microtome (Model: MU-290, Navyug). These sections were processed through standard hematoxylin and eosin (H & E) staining procedures and viewed under the microscope (Leica DM 750 microscope).

#### 2.12.1 Johnsen's score

To determine the Johnsen score of the testis, we applied the criteria established by Johnsen (1970), which uses a ten-point system to assess spermatogenesis based on cell profiles in the seminiferous tubules. We examined 30 seminiferous tubules per animal under a 100× magnification microscope. Each tubule was scored from 1 to 10, with 10 indicating the highest spermatogenesis activity and 1 indicating no germ cells. We categorized the scores into four groups: 1–3 (no germ cells or only spermatogonia), 4–5 (spermatocytes), 6–7 (spermatids), and 8–10 (mature sperm) [33].

### 2.13 Toxicity analysis

#### 2.13.1 Genotoxicity analysis

Immediately after cardiac puncture, blood smear was made on clean glass slides, preserved in absolute methanol for 3 min, and then allowed to air dry. For each group, five slides were prepared for each treated and untreated animals. The slides were stained with 10 % May-Grunwald-Giemsa stain, and MN frequency was measured by a single observer in coded slides. A total of 1000 erythrocytes (500 from each slide) from each mice were examined for the presence of MN at a 100× magnification under oil immersion microscope [34].

#### 2.13.2 Relative organ weight and toxicity marker analysis

After dissecting out the organs, the relative weight of kidney and liver were measured using a high-precision electronic balance (Model: PGB200, Wensar) and compared with the control groups.

Toxicity marker enzymes such as SGOT, SGPT, and creatinine level were analyzed following the standard protocol of the International Federation of Clinical Chemistry (IFCC). The limits of detection for test kits were 3 IU/L for SGOT, 5 IU/L for SGPT, and 0.1 mg/dl for creatinine.

#### 2.13.3 Histopathological analysis of liver and kidney

Histological studies of liver and kidney was performed similar to the methodology described for the histological analysis of testis.

### 2.14 Statistical analysis

Statistical analysis was performed using SPSS software (version 20) and Graph Pad Prism (version 5.0). The Kolmogorov-Smirnov (KS) test was used to determine the normality of the data distribution. The continuous data were reported as Mean ± Standard Error. To compare data among groups, the analysis of variance (ANOVA) test was used, followed by the post hoc Tukey test. P < 0.05 was chosen as the degree of significance.

## 3 Results

### 3.1 Water parameters

In the current investigation, the physicochemical properties and heavy metals of the water sample collected from the sampling sites were estimated. All the results of physicochemical parameters and heavy metal values of water samples were compared with WHO [35] and BIS [36] (Table 2).

**Table 2:** Concentrations of physiochemical properties of water and heavy metals in the water sample of Deepor Beel near Boragaon Garbage dumpyard during winter (December) and summer (July) season and their mean is presented below.

Parameters	Present Study	Proportion in the LCS	WHO [35]	BIS [36]
**pH**	10.92 ± 0.11 (8.14 ± 0.03–13.7 ± 0.19)	9.34 ± 0.03	6.5–9.5	6.5–8.5
**EC (μScm**^**−**^**^1^)**	4955 ± 65 (1170 ± 10–8740 ± 120)	1470 ± 40	500	–
**Turbidity (NTU)**	353 ±0 .091 (12.2 ± 0.58–693.8 ± 1.24)	67.2 ± 0.88	5	5
**DO (mg/L)**	5.81 ± 0.04 (2 ± 0.02–9.62 ± 0.06)	5.4 ± 0.04	–	–
**SO**_**4**_^**2−**^**(mg/L)**	191.46 ± 0.62 (0.137 ± 0.002–382.78 ± 1.22)	13.7 ± 0.22	250	200
**PO**_**4**_^**3−**^**(mg/L)**	14.645 ± 0.05 (9.770.02 ± 0.001–19.52 ± 0.09)	8.2 ± 0.49	–	–
**NO**_**3**_^**−**^ (**mg/L)**	38.915 ± 0.485 4.91 ± 0.02–72.92 ± 0.95	31.5 ± 1.02	50	45
**BOD (mg/L)**	234.75 ± 1 (10.9 ± 0.12–458.6 ± 1.86)	70.9 ± 1.5	–	–
**Na**^**+**^**(mg/L)**	98.22 ± 1.17 (21.4 ± 1.03–175.04 ± 1.30)	51.4 ± 2.4	200	–
**K**^**+**^**(mg/L)**	29.96 ± 0.17 (6.02 ± 0.10–53.9 ± 0.24)	21.2 ± 1.19	20	–
**Cl**^**−**^ (**mg/L)**	376.73 ± 2.1 (251.18 ± 1.34–502.28 ± 2.96)	259.48 ± 2.34	250	200
**F**^**−**^**(mg/L)**	1.185 ± 0.015 (0.25 ± 0.01–2.12 ± 0.02)	1.51 ± 0.061	1.5	–
**TDS (mg/L)**	460.90 ± 1.12 (4.39 ± 0.01–917.4 ± 2.23)	504 ± 2.23	500	–
**Al (mg/L)**	3.62 ± 0.05 (0.019 ± 0.003–7.21 ± 0.06)	1.59 ± 0.009	0.1–0.2	–
**As (μg/L)**	69.52 ± 0.125 (9.614 ± 0.03–129.42 ± 0.22)	31.614 ± 0.92	0.01	–
**Be (mg/L)**	0.014 ± 0.0008 (0.004 ± 0.0006–0.024 ± 0.001)	0.024 ± 0.004	0.012	–
**Cd (mg/L)**	BDL	BDL	0.003	0
**Ca (mg/L)**	167.56 ± 0.27 (15.02 ± 0.09–320.1 ± 0.44)	212 ± 2.44	200	75
**Cr (mg/L)**	0.0377 ± 0.003 (0.0172 ± 0.002–0.0582 ± 0.0004)	0.0542 ± 0.0008	0.05	0.05
**Cu (mg/L)**	0.0269 ± 0.0055 (0.0086 ± 0.001–0.0452 ± 0.001)	0.0472 ± 0.003	2	0.5
**Fe (mg/L)**	28.49 ± 0.405 (0.749 ± 0.01–56.23 ± 0.8)	6.749 ± 0.88	0.2–0.3	0.3
**Mg (mg/L)**	26.84 ± 0.245 (2.315 ± 0.13–51.36 ± 0.36)	22.615 ± 0.96	150	30
**Mn (mg/L)**	0.64 ± 0.01 (0.41 ± 0.01–0.87 ± 0.01)	0.68 ± 0.03	0.08	0.1
**Ni (mg/L)**	0.083 ± 0.002 (0.064 ± 0.002–0.102 ± 0.002)	0.094 ± 0.008	–	0.02
**Pb (mg/L)**	0.0222 ± 0.0015 (0.012 ± 0.002–0.0324 ± 0.001)	0.0224 ± 0.003	0.01	0.01
**Zn (mg/L)**	0.0705 ± 0.045 (0.008 ± 0.001–0.133 ± 0.09)	0.088 ± 0.003	0.01–0.05	–

In the table, all values are expressed in Mean ± SE. BIS refers to Bureau of Indian Standards, BDL- Below detection level, WHO- World Health Organization, DO- Dissolved Oxygen, EC- Electronic Conductivity, BOD- Biochemical Oxygen Demand, TDS- Total dissolved Solid, Al- Aluminum, SO_4_^2-^- Sulphate, PO_4_^3-^- Phosphate, NO_3_^−^ - Nitrate, F^−^- Fluoride, As- Arsenic, Be- Beryllium, Cd- Cadmium, Ca- Calcium, Cr- Chromium, Cu- Copper, Fe- Iron, Mg- Magnesium, Mn- Manganese, Ni- Nickel, Pb- Lead, Zn- Zinc.

Results revealed that pH, EC, Turbidity, DO, Sulphate, Phosphate, Nitrate, BOD, Potassium, Chloride, Fluoride, TDS, and heavy metals-aluminum (Al), arsenic (As), beryllium (Be), calcium (Ca), chromium (Cr), iron (Fe), manganese (Mn), magnesium (Mg), nickel (Ni), lead (Pb), and zinc (Zn) in LCS were higher than the permissible limit as recommended by WHO [35] and BIS [36].

### 3.2 Relative testicular weight

**Fig. 1:**
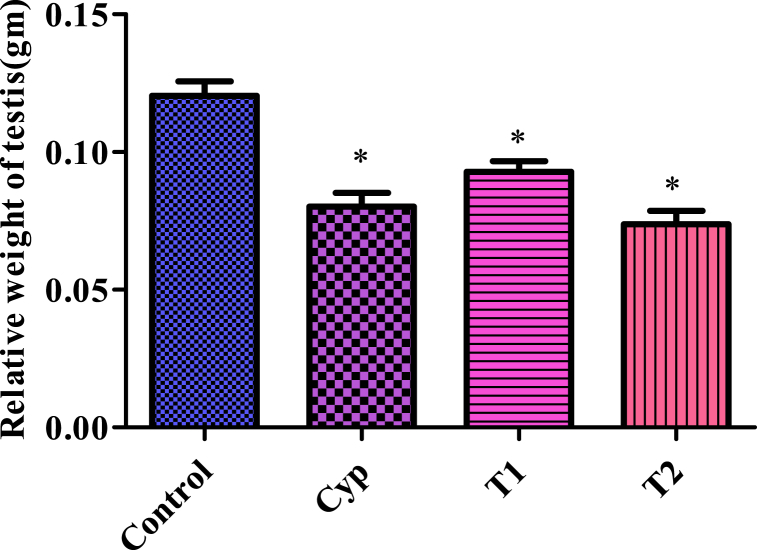
The changes of relative testicular weight in animals treated in Control, Cyp, T1 and T2 treated groups. All values are expressed in Mean ± SE. ∗ Signifies significant difference (P < 0.05) from the control group. In the figure, Cyp denotes Cyclophosphamide treated group, T1- Treatment group 1 (30 % LCS), T2- Treatment group 2 (70 % LCS).

After treatment period, we assessed the relative weight of the testes. It was observed that the treated groups exhibited a significant decrease (P < 0.05) in the relative testis weight compared to the control group following the administration of LCS. This decrease in testis weight showed a concentration-dependent pattern, with higher LCS concentrations resulting in more pronounced reductions in testis weight. Notably, Cyp treatment also led to a significant decrease in the relative testis weight, nearly equivalent to the weight observed in the T2 group (Fig. 1).

### 3.3 Sperm characteristics

#### 3.3.1 Sperm count

In this study, following administration of LCS, a significant decrease (P < 0.05) in spermatozoa count was observed in both T1 and T2 groups compared to the control group. This decrease in sperm count exhibited a concentration-dependent pattern, with the T2 group displaying the lowest value among all treatment groups. While the reduction in sperm count was more pronounced in the T2 group compared to the Cyp group, this difference did not reach statistical significance. Notably, there was a significant difference (P < 0.05) in sperm count between the T1 and T2 groups (Table 3).

**Table 3:** Total sperm count, sperm viability, and normal acrosome in control and treated groups.

Treatments	Sperm count × 10^6^	Sperm viability %	Normal Acrosome %
**Control**	379 ± 8.59	88 ± 2.55	79.6 ± 3.4
**Cyp**	241.8 ± 16.81^a^	57 ± 3.17^a^	48.2 ± 3.327^a^
**T1**	301.6 ± 4.007^ab^	72 ± 2.30^ab^	60.2 ± 3.15^a^
**T2**	227.4 ± 8.066^ac^	50.6 ± 5.66^ac^	43.8 ± 3.96^ac^

All values are expressed in Mean ± SE. In the Table, Cyp denotes Cyclophosphamide treated group, T1- Treatment group 1 (30 % LCS), T2- Treatment group 2 (70 % LCS). Superscript a-statistical significance (P < 0.05) from the control group. b-statistical significance (P < 0.05) from the Cyp group. c-statistical significance (P < 0.05) from the T1 group.

#### 3.3.2 Sperm viability

The sperm viability analysis showed that both T1 and T2 groups had significantly lower levels of viable sperm compared to the control group after LCS administration (P < 0.05). This decline in sperm viability was directly related to the concentration of LCS, with the T2 group displaying a more pronounced decrease in viability than the T1 group. Moreover, compared to the Cyp group, the reduction in sperm viability in the T2 group was even greater, although this reduction did not reach statistical significance. Notably, there was a significant difference in sperm viability between both the T1 and T2 groups (Table 3).

#### 3.3.3 Normal acrosome

In this study, the evaluation of acrosome demonstrated a notable decrease (P < 0.05) in the number of normal acrosome sperms in both T1 and T2 groups treated with LCS compared to the control group. Furthermore, T2 treatment exhibited a more substantial reduction in intact acrosome sperm compared to the group treated with Cyp, although this difference did not reach statistical significance. Notably, in terms of acrosome integrity, both LCS-treated groups (T1 and T2) displayed significant differences (P < 0.05) (Table 3).

#### 3.3.4 Sperm morphology

**Fig. 2:**
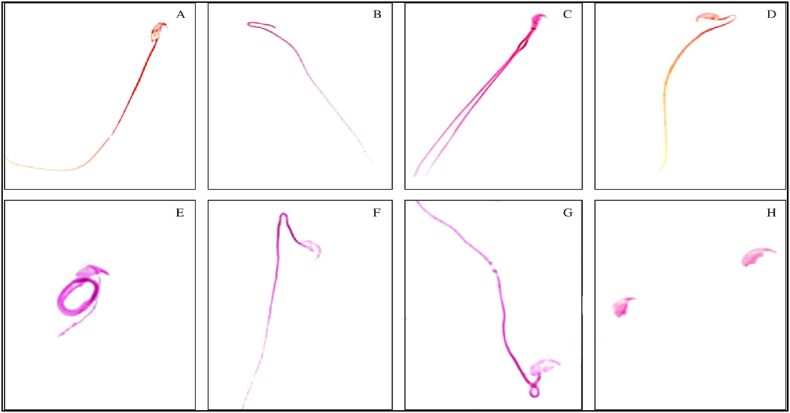
Representative photomicrographs showing sperm morphologies (tail) observed from control and treated groups. The sperms were stained by eosin. A) normal sperm cell, B) headless, C) double tailed, D) bent neck, E) coiled tail, F) bent tail, G) looped tail, H) tailless. Magnfication 100 × .

**Fig. 3:**
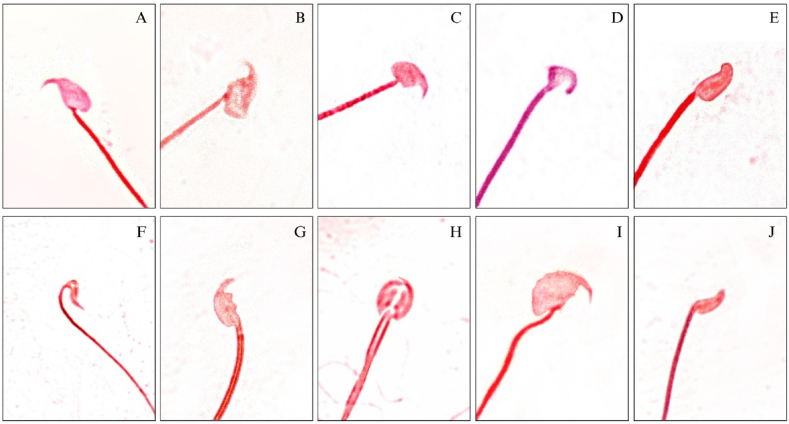
Representative photomicrograph of sperm head abnormalies observed in sperm abnormality assay: A: Normal sperm head; B: Amorphous head; C: Beaked; D: Hooked; E: Hookless; F: Pin headed; G: Banana shaped; H: Double headed; I: Giant and J: Dwarf headed. Sperms were stained by Eosin. Magnification 100 × .

LCS administration revealed several defects in sperm heads and tails (Fig. 2, Fig. 3). Defective sperm morphology was significantly higher in both LCS-treated groups, T1 and T2, compared to the control group. The defects increased in a concentration-dependent manner, with T2 showing significantly more defects than T1. Additionally, when compared to the Cyp group, the T2 group exhibited more pronounced sperm defects, although the difference was not statistically significant (P < 0.05) (Table 4, Table 5).

**Table 4:** Sperm morphological abnormality in animals treated with control and LCS.

Treatment	Average % abnormal Sperm tail abnormalities	Types of sperm tail abnormalities (in %)						
		Headless	Double tailed	Bent neck	Coiled tailed	Bent tail	looped	tailless
Control	4.5 ± 0.22	1.3	0.1	0.5	0.2	0.6	0.4	1.4
Cyp	42.7 ± 3.75^a^	8.0	6.6	6.0	6.7	4.7	4.2	6.5
T1	14.30 ± 0.93^ab^	4.3	0.5	1.7	1.0	2.1	1.5	3.2
T2	49.10 ± 2.45^ac^	7.9	4.8	7.3	7.4	8.3	4.9	8.5

All values are expressed in Mean ± SE. In the Table, Cyp denotes Cyclophosphamide treated group, T1- Treatment group 1 (30 % LCS), T2- Treatment group 2 (70 % LCS). Superscript a-statistical significance (P < 0.05) from the control group. b-statistical significance (P < 0.05) from the Cyp group. c-statistical significance (P < 0.05) from the T1 group.

**Table 5:** Sperm head abnormality in mice treated with control and LCS.

Treatment	Average % abnormal Sperm head abnormalities	Types of sperm head abnormalities in %								
		Amorphous	Beaked	Hooked	Hook less	Pin headed	Double headed	Banana shaped	Giant	Dwarf
Control	4.6 ± 0.48	0.1	1.7	0.5	0.4	0.2	0.2	0.5	0.3	0.7
Cyp	41.5 ± 2.46^a^	1.6	11.6	3.0	7.2	2	4.6	3.8	3.6	4.5
T1	15.7 ± 0.96^ab^	0.6	4.9	1.6	1.8	0.5	2.1	2.1	1.1	1.0
T2	47.0 ± 2.31^ac^	2.2	11	3.0	5.0	3.7	5.8	6.0	5.2	5.1

All values are expressed in Mean ± SE. In the Table, Cyp denotes Cyclophosphamide treated group, T1- Treatment group 1 (30 % LCS), T2- Treatment group 2 (70 % LCS). Superscript a-statistical significance (P < 0.05) from the control group. b-statistical significance (P < 0.05) from the cyp group. c-statistical significance (P < 0.05) from the T1 group.

The examination identified seven sperm tail abnormalities: headless, double-tailed, bent neck, coiled tail, bent tail, looped tail, and tailless. In the Cyp group, the percentage of headless sperm was the highest, followed by sperm with coiled tails. In the T2 group, the highest percentage was of tailless sperm, followed by those with bent tails (Fig. 2A–H). Again, the examination of sperm head morphology unveiled the presence of nine distinct types of frequently observed sperm head defects, namely amorphous, hookless, hooked, beaked, banana-shaped, pin-headed, double-headed, giant, and dwarf. The percentage of beak-headed sperm was found to be the highest in all the treatment groups (Fig. 3A–J). Like the trends observed in sperm abnormalities, sperm head defects also exhibited a concentration-dependent increase. Notably, the T2 group displayed the highest incidence of sperm head abnormalities, as outlined in Table 5.

**Fig. 4:**
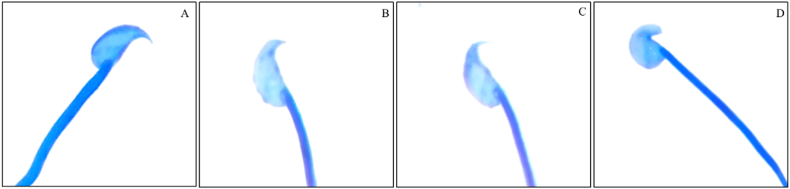
Representative photomicrograph of sperm with acrosome, (A) Control group, (B) Cyclophosphamide treated group, (C) T1 (30 % LCS treated group), (D) T2 (70 % LCS treated group). Sperms were stained by Commassie brilliant blue. Magnification 100 × . (For interpretation of the references to colour in this figure legend, the reader is referred to the Web version of this article.)

Acrosome analysis reveals that in the control group, the acrosome is intact. In the Cyp treated group, acrosome is absent. In the T1 group, acrosome is not distinct. Again, in the T2 group, acrosome is absent (Fig. 4A–D).

### 3.4 Assessment of the antioxidant defense system in testis

**Fig. 5:**
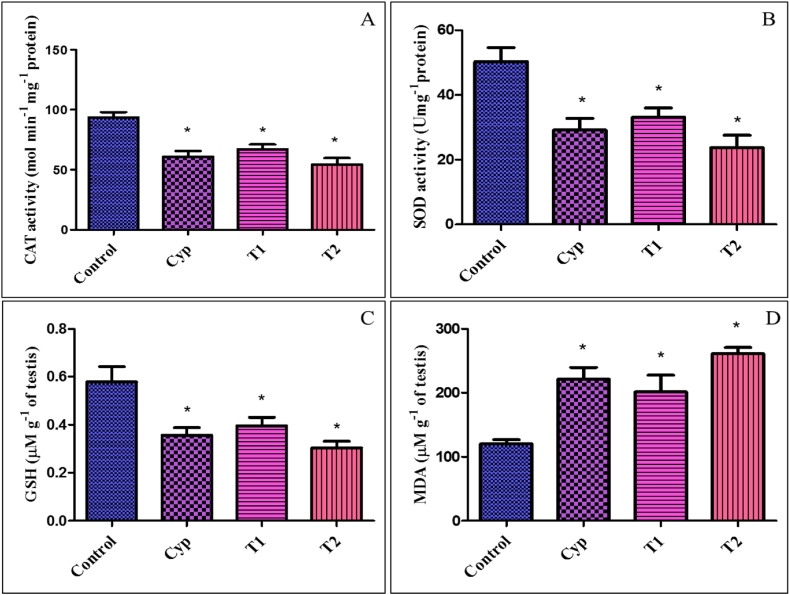
Stress indicators in the testicular homogenate- (A) Catalase (CAT), (B) Superoxide Dismutase (SOD), (C) Glutathione (GSH), and (D) Malondialdehyde (MDA). All values are expressed in Mean ± SE.∗ Signifies significant difference (P < 0.05) from the control group. In the figure, Cyp denotes Cyclophosphamide treated group, T1- Treatment group 1 (30 % LCS), T2- Treatment group 2 (70 % LCS), GSH- Glutathione, CAT- Catalase, SOD- Superoxide Dismutase.

After LCS treatment, an assessment of stress indicators in the testicular homogenate indicated a significant decrease (P < 0.05) in CAT and SOD activities, along with a reduction in GSH concentration, alongside an increase in MDA levels, within both the T1 and T2 groups in comparison to the control group. However, the magnitude of the decrease in CAT, SOD, and GSH levels, as well as the increase in MDA, was more prominent in the T2-treated group (Fig. 5A–D). The group treated with the standard drug, Cyp, also revealed a significant decrease in the case of CAT, SOD, and GSH and a significant increase of MDA compared to the control group. Furthermore, concerning all the stress biomarkers, neither of the LCS-treated groups (T1 and T2) exhibited significant differences (P < 0.05) when compared to the Cyp group (Fig. 5A–D).

### 3.5 Testosterone

**Fig. 6:**
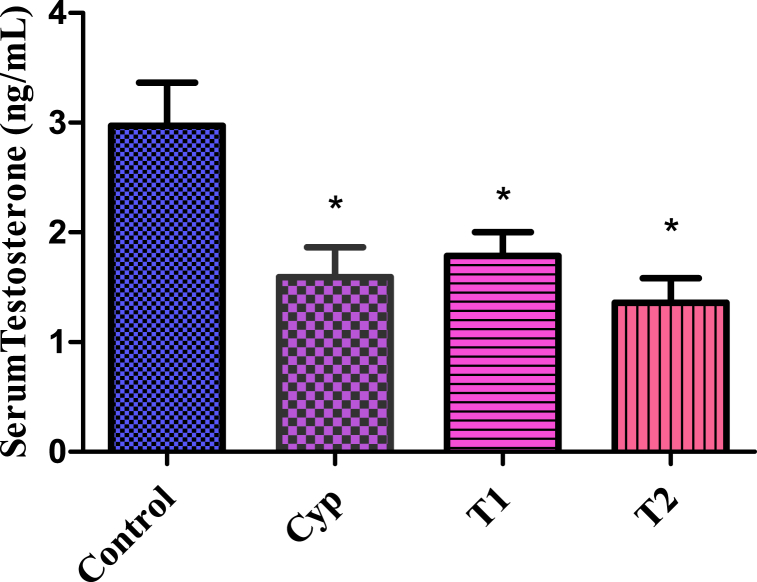
Serum testosterone levels between the treated groups. All values are expressed in Mean ± SE. ∗ Signifies significant difference (P < 0.05) from the control group. In the figure, Cyp denotes Cyclophosphamide treated group, T1- Treatment group 1 (30 % LCS), T2- Treatment group 2 (70 % LCS).

In this study, LCS administration led to a significant decrease (P < 0.05) in serum testosterone levels in both T1 and T2 groups compared to the control group. Notably, the T2 group exhibited a more pronounced decline in testosterone levels. The group treated with the standard drug Cyp also demonstrated a significant decrease in testosterone concentration. Additionally, there were no significant differences (P < 0.05) in terms of serum testosterone levels between the treated groups (Fig. 6).

### 3.6 Histopathological study of testis

**Fig. 7:**
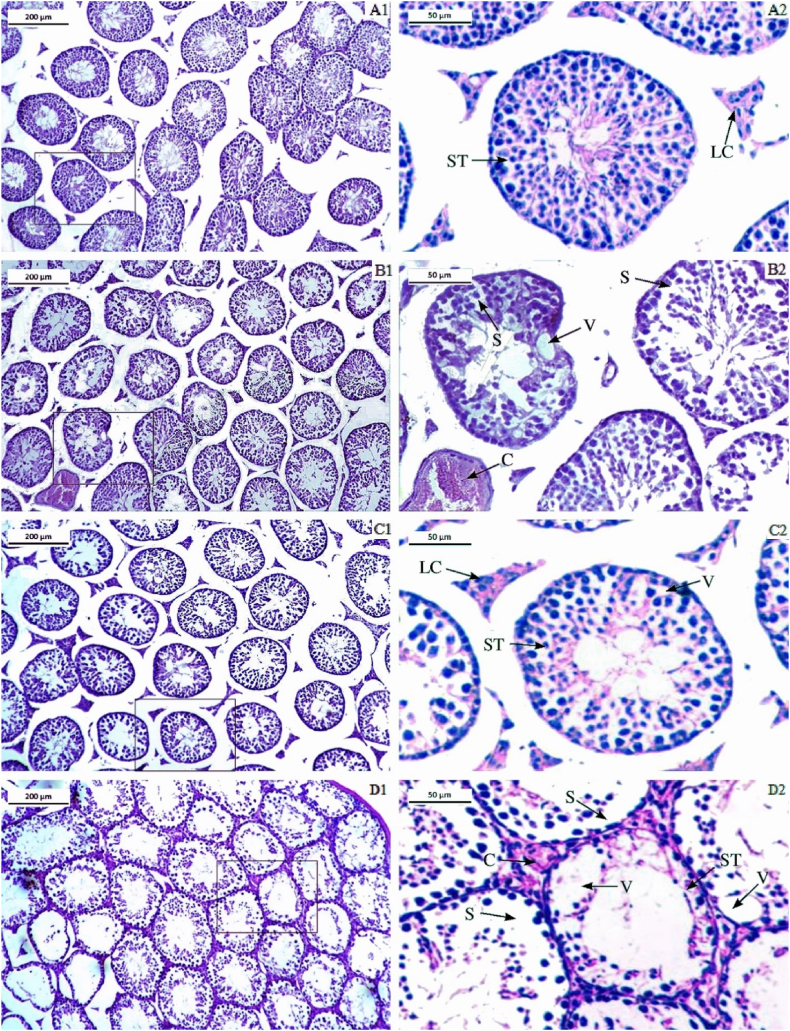
Photomicrographs of histoarchitecture of testis from different treated groups- A1 and A2-control group, B1 and B2- Cyclophosphamide treated group, C1 and C2- LCS treated group T1, and D1 and D2- LCS treated group T2. In the figure the abbreviations ST represents the seminiferous tubule; SC represents the Sertoli cell; LC represents - Leydig cell; V represents-vacuole; S- represents Sloughing of cells; C represents blood congestion. Stain- Hematoxylin and Eosin. Magnification- 10 × - scale bar 200 μm and 40 × - scale bar 50 μm.

Photomicrographs of the H&E stained testis sections from the control group showed normal histoarchitecture of seminiferous tubules with regular spermatogenesis under microscopic analysis. Within the seminiferous tubules, the Sertoli and germ cells were both normal and no histological lesions were observed (Fig. 7, A1-A2). While the treated group showed, structural disturbances in the testicular sections of the LCS treated mice. However, the severity of the histological changes varied between the LCS-treated groups (T1 and T2). Compared to the testis sections from the control group, the treated (T1 and T2) testis sections showed irregularities in seminiferous tubules and sperm quantity and loosely arranged spermatogonia in seminiferous tubules. Additionally, seminiferous tubules showed signs of vacuole formation, and cells could be seen sloughing into the lumen of the tubules (Fig. 7, C1-C2 and D1-D2). Similarly, in the Cyp-treated group, fewer sperm cells and irregular spermatogenesis were observed in the lumen of some seminiferous tubules as that of the T2 group (Fig. 7, B1-B2).

#### 3.6.1 Johnsen's score

**Fig. 8:**
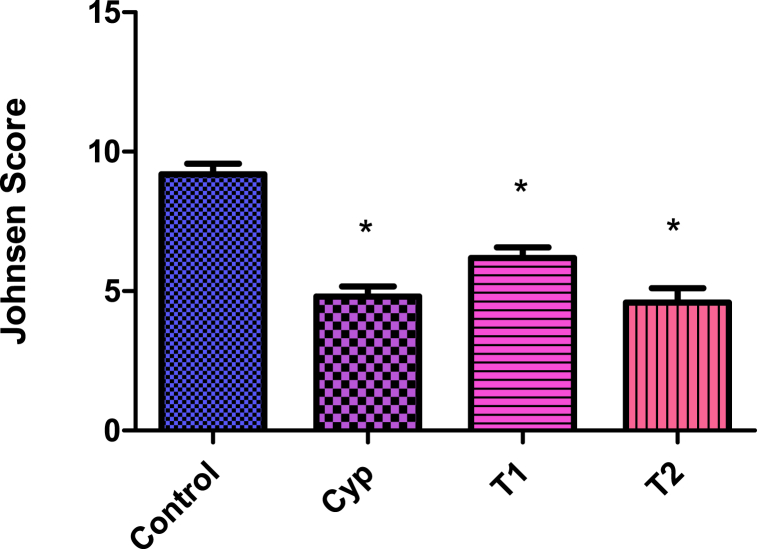
Johnsen's score of the testis in different treatment groups. All values are expressed in Mean ± SE. ∗ Signifies significant difference (P < 0.05) from the control group. In the figure, Cyp denotes Cyclophosphamide treated group, T1- Treatment group 1 (30 % LCS), T2- Treatment group 2 (70 % LCS).

The assessment of the Johnsen score revealed that the control group displayed normal testicular morphology, whereas the other three groups (Cyp, T1, and T2) showed significant testicular damage. The mean Johnsen scores were 9.2 ± 0.38 for the control group, 4.8 ± 0.37 for the Cyp group, 6.2 ± 0.4 for group T1, and 4.6 ± 0.05 for group T2. All treated groups had significantly lower scores compared to the control, with the Cyp and T2 treatments causing nearly equal testicular damage (Fig. 8).

### 3.7 Toxicity analysis

We studied genotoxicity and systemic toxicity. Under genotoxicity study we analyzed MN occurrence. Again, under systemic toxicity, we analyzed relative weight and histoarchitecture of liver and kidney and biomarkers of liver and kidney function.

#### 3.7.1 Frequency of MN in the RBC of treated and control mice

**Fig. 9:**
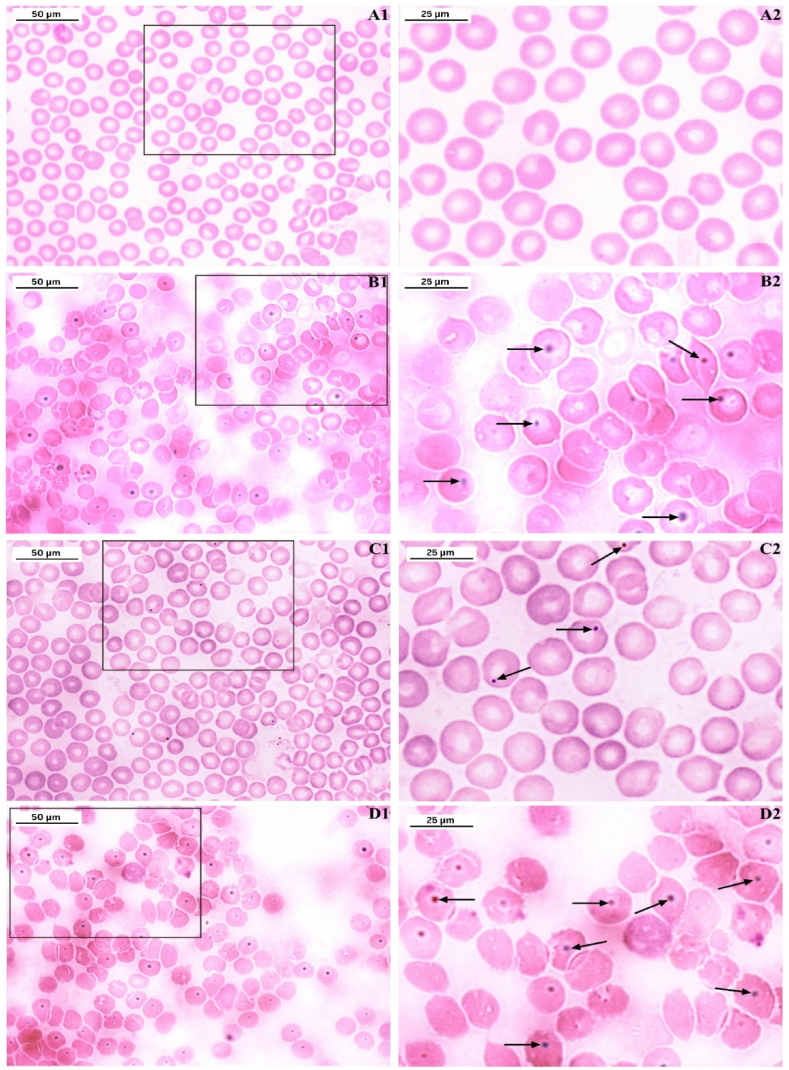
Photo-micrographic presentation of Giemsa stain of MN of control and treated RBC cells of albino mice. A1 and A2-control group, B1 and B2- Cyclophosphamide treated group, C1 and C2- LCS treated group T1, and D1 and D2- LCS treated group T2. Magnification 100 × .

The genotoxic effect was assessed using the MN assay. The assessment revealed that both the T1 and T2 groups experienced a significantly higher frequency of MN in their red blood cells (RBCs) compared to the control group (P < 0.05). This increase in MN exhibited a concentration-dependent pattern, with the T2 group showing a higher frequency of MN than the T1 group. Furthermore, compared to the Cyp-treated group, the T2 group had a greater number of MN, although this difference was not statistically significant. However, it is important to note that the frequency of MN in both the T1 and T2 groups differed significantly from one another (P < 0.05), as indicated in Table 6 and Fig. 9 (A1–D1, A2–D2).

**Table 6:** Relative weight of liver and kidney and enzyme activity of SGOT, SGPT, creatinine and the frequency of MN.

Groups	Relative Weight of Liver	Relative Weight of Kidney	SGOT (U/L)	SGPT (U/L)	Creatinine (U/L)	MN (Frequency)
**Control**	1.64 ± 0.059	0.26 ± 0.009	61.91 ± 2.541	26.83 ± 0.970	0.42 ± 0.049	0.09 ± 0.008
**Cyp**	1.28 ± 0.045^a^	0.20 ± 0.007^a^	228.60 ± 8.823^a^	67.59 ± 4.469^a^	1.08 ± 0.093^a^	7.79 ± 0.098^a^
**T1**	1.39 ± 0.058^a^	0.19 ± 0.009^a^	158.0 ± 6.470^ab^	46.90 ± 4.664^ab^	0.82 ± 0.026^a^	3.94 ± 0.115^ab^
**T2**	1.26 ± 0.057^a^	0.21 ± 0.006^a^	242.3 ± 8.032^ac^	72.20 ± 2.755^ac^	1.40 ± 0.132^ac^	7.83 ± 0.308^ac^

All values are expressed in Mean ± SE. MN are indicated as occurrence (frequency, %) in blood erythrocytes. In the Table, Cyp denotes Cyclophosphamide treated group, T1- Treatment group 1 (30 % LCS), T2- Treatment group 2 (70 % LCS), SGOT- Serum Glutamic-Oxaloacetic Transaminase, SGPT- Serum glutamic pyruvic transaminase. Superscript a-statistical significance (P < 0.05) from the control group. b- Statistical significance (P < 0.05) from the cyp group. c- Statistical significance (P < 0.05) from the T1 group.

#### 3.7.2 Relative organ weight and the marker enzymes (SGOT, SGPT, and creatinine)

After administration of LCS, a notable reduction in the relative weight of the liver and kidney was observed, with statistical significance (P < 0.05) compared to the control group. This decrease in organ weight exhibited a concentration-dependent trend, where higher doses of LCS treatment led to more substantial reductions in organ weight. Remarkably, the group receiving the highest dose of LCS treatment, labeled as T2, showed the lowest organ weights among all the treatment groups. Compared to the Cyp (Control) group, the reduction in organ weight within the T2 group was more pronounced, though it did not reach statistical significance (Table 6).

Following treatment period, a significant increase in the levels of SGOT, SGPT, and creatinine was observed in the T1 and T2 groups compared to the control group (Table 6). These toxicity biomarker levels demonstrated variation dependent on the treatment concentration, with lower concentrations resulting in milder increases and higher concentrations causing more pronounced elevations. Specifically, the levels of SGOT and SGPT in the T1 group exhibited a statistically significant difference (P < 0.05) when compared to the Cyp group. However, the creatinine level in the T1 group did not show a significant difference from the Cyp-treated group. In contrast, the levels of SGOT, SGPT, and creatinine in the Cyp and T2 groups remained nearly similar, with no statistically significant differences. Notably, a significant difference (P < 0.05) in the levels of SGOT, SGPT, and creatinine was observed between the T1 and T2 groups (Table 6).

#### 3.7.3 Histoarchitecture of liver and kidney

**Fig. 10:**
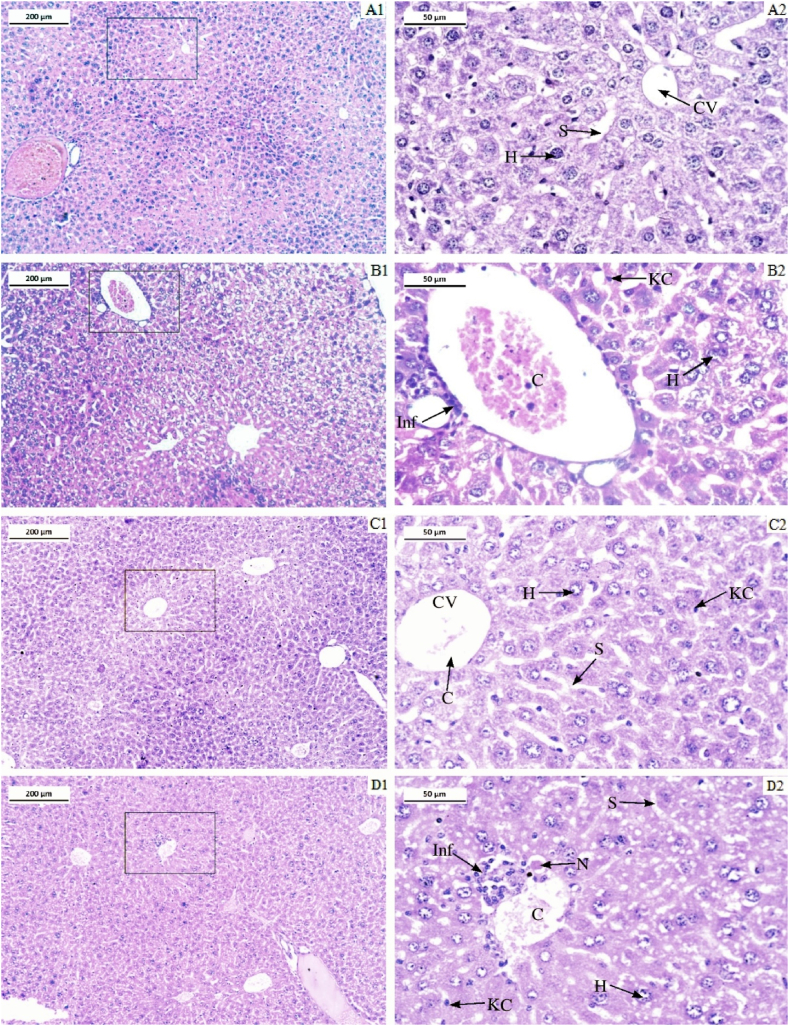
Photomicrograph of Liver sections. A1 and A2-control group, B1 and B2- Cyclophosphamide treated group, C1 and C2- LCS treated group T1, and D1 and D2- LCS treated group T2. In the figure- CV represents central vein, S represents sinusoids, H represents hepatocytes and KC represents Kupffer cell, Inf represents infiltrations of inflammatory cells, N represents Necrosis. Stain- Hematoxylin and Eosin. Magnification- 10 × , scale bar 200 μm and 40 × , scale bar 50 μm.

In the present study, the liver section from the control group revealed that the histoarchitecture of the liver cells was without any lesions, with hepatocytes radiating from the central vein (Fig. 10, A1 - A2). However, in LCS-treated groups T1 and T2, severe congestion of the portal vein of the hepatic triad was noticed, accompanied by mass infiltration of inflammatory cells, the proliferation of Kupffer cells, hyperplasia of the portal vein, with discrete hemorrhagic areas (Fig. 10, C1–C2 and D1–D2). Moreover, the T2 group also experienced single-cell necrosis, characterized by a spherical, shrunken hepatocyte surrounded by a distinct halo. All the lesions observed in the treated groups were similar to the Cyp-treated group. However, histology shows that the T2 group suffered more severe damage than the Cyp group (Fig. 10, B1–B2).

**Fig. 11:**
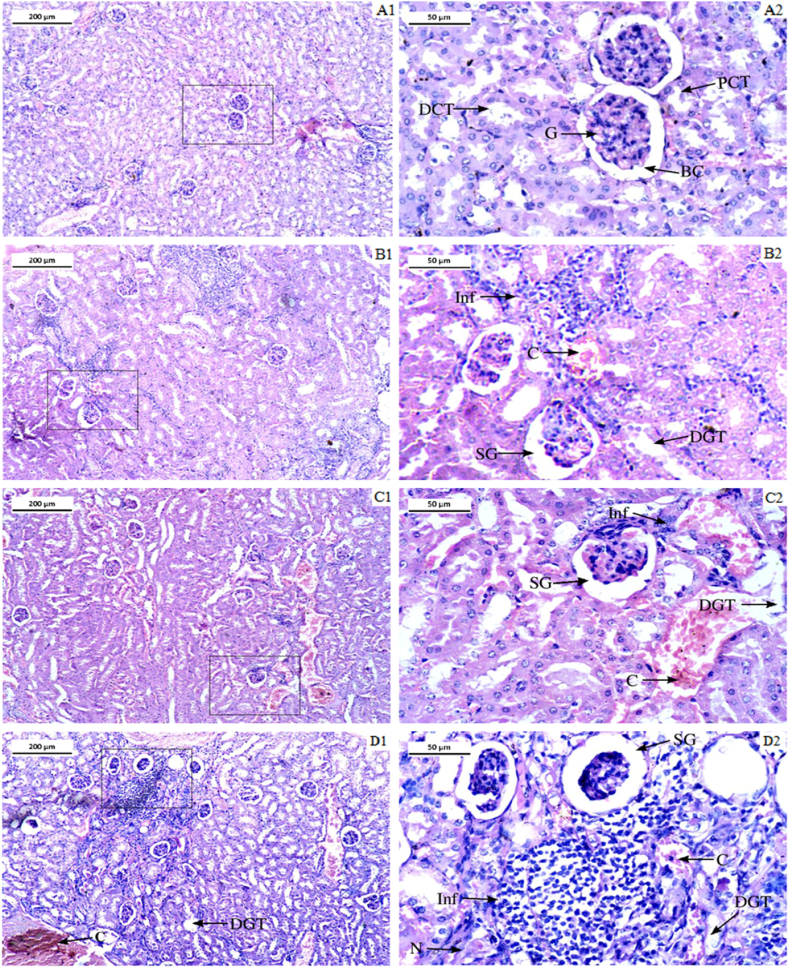
Photomicrograph of kidney sections. A1 and A2-control group, B1 and B2- Cyclophosphamide treated group, C1 and C2- LCS treated group T1, and D1 and D2- LCS treated group T2. In the figure- G represent glomerulus, DGT represents tubular degeneration, Inf represents infiltrations of inflammatory cells, C represents Congestion, SG-reprsents shrinkage of glomerulus, N represents necrosis, PCT represents the proximal covulated tubule; DCT represents the distal covulated tubule. Stain- Hematoxylin and Eosin Magnification- 10 × , scale bar 200 μm and 40 × , scale bar 50 μm.

The kidney section from the control group had characterized renal tubules and glomeruli (Fig. 11, A1–A2). The high level of renal tubular degeneration (DGT), renal tubular cell necrosis (N), and significant infiltration of inflammatory cells (Inf) were evident in the kidney sections of the LCS-treated groups (T1 and T2). Additionally, in the T2 group, glomerular shrinkage (SG) and congestion (C) were seen (Fig. 11, C1–C2 and D1–D2). The type of degenerations of renal tissue and glomerular shrinkage observed in the Cyp group was similar to the LCS-treated group. The T2 group experienced the most severe damage among all the treated groups (Fig. 11, B1–B2).

## 4 Discussion

Male fertility depends on sperm quality, count per ejaculation, normal sperm morphology, intact acrosome, and healthy testis histoarchitecture. Changes in these parameters can significantly impact male reproduction. Reports indicate that leachate contamination can lead to reproductive toxicity, often resulting in infertility, along with genotoxicity and systemic toxicity [[12], [13], [14]]. Therefore, this investigation assessed whether leachate from the Baragaon dumpyard induces reproductive toxicity, as well as genotoxicity and systemic toxicity.

The results of this study revealed altered physicochemical parameters and heavy metal concentrations exceeding recommended limits [35,36], indicating pollution of the Deepor Beel water from sewage disposal. The more alkaline pH suggests that the dump yard is maturing under aerobic conditions. The high concentration of dissolved organic materials, such as ionically charged organic acids, significantly contributes to the total alkalinity. Combined with the increased level of TDS, this alkalinity imparts an unfavorable taste to the water, which is detrimental to human health [9]. The increase in EC levels may be attributed to the higher amounts of ions present in the samples [37]. The increased turbidity in the LCS reflects the higher concentration of suspended particles in the water. The low DO levels indicate poor water quality, while the elevated BOD levels are likely due to the presence of organic compounds in the LCS [9]. The increase in Cl^−^ and NO_3_^−^ levels, which are indicators of water pollution, suggests severe pollution in the LCS. Higher concentrations of Na^+^, K^+^, and Zn in the water may result from the dissolution of soil salts or evaporation of salts due to waste dumping [37]. The rise in Pb and other heavy metals likely stems from the dumping of industrial waste, vehicular waste, and waste batteries [38]. These findings align with those reported by Baawain et al. [39] and Alimba et al. [5].

The testis is the factory for sperm production. Changes in the weight of the testis adversely affect sperm production. In this study, LCS administration significantly reduced relative testicular weight in the T1 and T2 groups compared to the control. The regulation and functioning of the testis are under the control of the principal androgen, testosterone [40]. The LCS-treated mice experienced low serum testosterone levels compared to the control group. This lowered testosterone level might be linked to the reduction in the testicular weight [41]. Moreover, the mass of the differentiated spermatozoa plays an important part in determining the weight of the testes [40]. Thus, the reduced number of germ cells may be the cause of the decline in the relative weight of the testes [42].

Adequate sperm production is necessary for successful fertilization. Lower production of sperm leads to infertility. The sperm count results revealed a significant decrease in epididymal sperm in both treated groups, possibly due to the heavy metals in the leachate. Pb reduces the quantity of Sertoli cells that are pivotal for spermatogenesis [43]. Heavy metals also reduce the ability of the testis to produce steroids, leading to lower testosterone levels and decreased sperm production [44]. Other heavy metals such as Al, As, Cr, Fe, and Mn also lower sperm count [[45], [46], [47]]. Disruption of the antioxidant defense system is a key mechanism by which Zn and Ni interfere with spermatogenesis [48,49]. Oxidative degradation resulting in disordered spermatogenesis and steroidogenesis has been extensively reported in earlier literature [45]. Heavy metals induce ROS formation. While a physiological concentration of ROS is necessary for spermatogenesis, excessive ROS becomes toxic and poses significant health risks, including infertility [50]. Excessive ROS causes Sertoli cell apoptosis, and ROS and oxidative stress are key mechanisms of reproductive toxicity [51]. Fewer or absent Sertoli cells prevent spermatogonia from maturing into sperm, leading to reduced sperm count.

Spermatozoa are produced from spermatogonia through mitosis, meiosis, and spermiogenesis. Hormonal changes, blood-testis barrier (BTB) impairment, or disruptions in spermatid formation due to harmful substances can lead to morphological imperfections [52]. Normal sperm morphology is crucial for fertilization and is a strong predictor of male sexual potency [53]. In this study, the LCS groups exhibited abnormal sperm morphologies, including defective heads and tails. Beaked-headed sperm were most prevalent in both LCS-treated groups. Various tail abnormalities were observed, such as double tails, bent necks, coiled tails, bent tails, looped tails, and tailless sperm. These varied abnormalities suggest that leachate components severely impact sperm [12]. Heavy metals in LCS may have disrupted the pre-meiotic DNA synthesis phase, leading to sperm abnormalities [54]. For instance, Pb induces abnormal sperm populations [55], while Ni, As, and Al are known to damage sperm morphology [56,57]. Additionally, oxidative damage and lipid peroxidation induced by these metals likely exacerbated sperm morphology.

Viable sperm are essential for successful fertilization. Exposure to hazardous chemicals typically decreases sperm viability [4,5]. The current study found that the LCS groups had significantly fewer viable sperm compared to the control group. Heavy metals in the leachate may have increased DNA fragmentation, resulting in reduced sperm viability [4,5]. These findings align with earlier studies [58,59] that highlighted the detrimental effects of heavy metal toxicity on sperm viability. The lower number of viable sperm in the LCS group may be due to LCS's adverse effects on the anterior pituitary, which produces LH and FSH. FSH and testosterone work synergistically to convert spermatids into spermatozoa; when this conversion is disrupted, spermatids die. Thus, the reduction in viable sperm may result from interference with the gonadotropin-producing regions of the anterior pituitary [60]. Additionally, FSH is crucial for the development and maintenance of Sertoli cells. A decrease in FSH may lead to fewer Sertoli cells, further reducing viable spermatozoa [60].

The acrosome is crucial for fertilization as its exocytosis releases enzymes that facilitate sperm entry into the ovum. Analysis revealed acrosome loss in the LCS group, likely due to heavy metals causing premature exocytosis of acrosomal vesicles. These findings align with past research indicating that heavy metals such as Pb, Fe, and Mn induce early acrosomal exocytosis, leading to infertility [61,62]. H_2_O_2_, a type of ROS, can lead to premature sperm hyperactivation and acrosome reaction. Heavy metals are also known to inhibit capacitation, which is essential for sperm's penetrative ability. Without proper capacitation, sperm cannot undergo chemotactic movement towards the ovum, significantly reducing the number of competent spermatozoa reaching the ovum [41,[60], [61], [62]].

The steroidogenesis pathway relies on three key enzymes—CYP11A1, StAR, and 17β-HSD that convert cholesterol to testosterone, crucial for testosterone production and release [63]. Accumulation of heavy metals affects these enzymes, significantly reducing the production of testosterone [64]. This study confirms the presence of heavy metals in leachate, which likely impacted the steroidogenesis pathway and suppressed testosterone production [6,42]. The synthesis and secretion of testosterone largely depend on LH stimulation of Leydig cells. However, the presence of heavy metals disrupts the hypothalamic-pituitary-gonadal axis, leading to reduced testosterone production [60,65]. Oxidative stress further damages Leydig cells through lipid peroxidation, mitochondrial dysfunction, and apoptosis, severely impairing testosterone production [66]. These effects prevent LH from effectively binding to Leydig cells. Any one, or a combination, of these mechanisms may be responsible for the lower testosterone levels observed.

Oxidative stress arises from excessive ROS production coupled with an impaired antioxidant defense system. Sperm cells are particularly vulnerable to oxidative stress due to the high concentration of polyunsaturated fatty acids in their biomembranes, making them prone to lipid peroxidation [67]. In this study, oxidative stress was indicated by elevated MDA levels and a decrease in antioxidant biomarkers in the testis. Heavy metal exposure often generates ROS such as OH^−^•, O2^-^•, H_2_O_2_, and lipid radicals, which disrupt the antioxidant defense system, leading to oxidative stress. Antioxidant enzymes like GSH, SOD, and CAT are crucial in scavenging ROS. However, heavy metals such as lead, arsenic, and mercury strongly bind to the thiol (-SH) or selenol (SeH) groups of GSH and SOD, compromising their integrity and reducing their levels [68,69]. SOD serves as the first line of defense against ROS by catalyzing the conversion of superoxide into H_2_O_2_ and water. During heavy metal toxicity, SOD levels decrease due to interactions between heavy metals and the metallic components of SOD (Zn, Mn, and Cu), inhibiting SOD and reducing its concentration [70,71]. This inhibition leads to an accumulation of superoxide radicals, which can deactivate CAT [72]. The activity of glutathione peroxidase, which is essential for hydrolyzing H_2_O_2_, depends heavily on GSH levels. GSH, composed of cysteine, glutamic acid, and glycine, is reduced when heavy metals like cadmium bind to its cysteine residue, thereby inactivating glutathione peroxidase [70]. Lipid peroxidation, an uncontrolled process, occurs continuously in the body but accelerates significantly under increased oxidative stress [73].

Histology is a fundamental microscopic technique used to distinguish cellular changes in treated tissue from that of a control group. Histological examination of the testes revealed testicular pathology, including sloughing of cells, vacuole formation, a reduced number of mature sperms, and loosely arranged spermatogonia in both LCS-treated groups. The oxidative stress generated may have contributed to these observed histological changes [57,74]. The loose arrangement of spermatogonia likely results from the death of spermatogonial cells due to oxidative stress [75]. Additionally, the vacuoles may have formed due to apoptosis of Sertoli or germ cells induced by oxidative stress [75].

MN form alongside the primary nucleus when acentric fragments or lagging chromosomes fail to integrate into one of the daughter nuclei during cell division [76]. This study revealed a significant increase in MN in the RBCs of LCS-treated mice. These findings align with Adeoye et al. [76], who reported that heavy metals in pharmaceutical effluents cause DNA damage and increase genomic instability in male rats. This DNA damage and instability may result from heavy metal accumulation, which interacts with biomolecules such as DNA, RNA, or proteins, leading to fragmentation. The generated free radicals further fragment DNA and RNA, with the fragments accumulating in the cytoplasm to form MN [11,61]. Therefore, it can be inferred that DNA fragmentation, and the accompanying free radical production, leads to an increase in MN [77]. MN can also form due to the failure to segregate chromosomes during anaphase, a condition that arises when heavy metals disrupt cell cycle control machinery. Heavy metals can affect different stages of the cell cycle, leading to arrest or sometimes bypassing control systems. For instance, lead (Pb) has been reported to interfere with cell cycle checkpoints, resulting in MN formation [11]. The findings in our study are consistent with reports that municipal effluent causes chromosome aberrations in bone marrow cells and cytotoxicity in multiple organs of Wistar rats [78].

The systemic toxicity assessment results showed a decrease in the weight of the liver and kidney in both LCS-administered groups. These findings align with the histological observations in this study, which revealed pyknosis, vacuolar degeneration, inflammatory infiltrates, hepatic lobule impairment, severe hepatocyte necrosis, massive leucocyte infiltration, glomerular shrinkage, and necrosis of nephrons and tubules in these organs. Hepatic necrosis and cellular infiltrations with inflammatory cells observed in LCS-treated mice have also been reported in workers exposed to chemicals from industrial wastes, where the wastes contain hepatotoxic substances [79]. Similar conditions have been recorded in the kidneys of male rats due to lead (Pb) poisoning [13]. The cellular degeneration and inflammatory infiltrates in the liver and kidney of LCS-treated mice may contribute to the observed decrease in organ weight [14,80]. Proximal tubular cells are particularly vulnerable to toxic xenobiotics because they are the first renal epithelial cells exposed to filtered toxic compounds. Therefore, the degeneration of renal tubular epithelia and necrotic changes observed in leachate-treated rats indicate kidney damage [81]. Exposure to LCS led to abnormal elevations in SGOT, SGPT, and creatinine serum levels. Heavy metals may induce lipid peroxidation, altering the cell membrane's permeability and allowing SGOT and SGPT to seep out of the hepatocytes' cytoplasm into the bloodstream [82]. This seepage is further supported by the significant rise in serum MDA levels and hepatic cell degeneration observed in the treated animals. The elevation of serum creatinine concentration is typically associated with reduced renal function [83]. The increased serum creatinine levels observed in treated mice compared to the control group suggest potential renal dysfunction caused by the heavy metals in LCS effluent. These findings are consistent with reports that rats exposed to pharmaceutical effluent [76] and municipal landfill leachate [84] exhibited significantly elevated creatinine levels and the development of histopathological lesions. The generation of ROS may account for the hepatic and renal damage observed in LCS-treated mice. This explanation is supported by reports indicating that the heavy metals (Cu, Fe, Cd, Pb, Ni, Cr, Mn, and As) cause damage to the liver and kidneys through ROS formation [85]. The findings suggest that LCS causes severe reproductive toxicity, genotoxicity, and cytotoxicity due to heavy metals, highlighting the urgent need to address environmental contamination to protect public health.

Further research will be required to pinpoint which specific heavy metal in LCS severely affects reproductive, systemic, and genetic parameters.

## 5 Conclusion

In conclusion, the experimental findings confirmed the presence of heavy metals in the LCS, which induced reproductive, genetic, and cytotoxic effects in mice. Reproductive toxicity was evidenced by a notable reduction in testis weight, sperm count, sperm viability, normal sperm morphology, acrosome integrity, and serum testosterone concentration. Genotoxicity was marked by the appearance of MN. Systemic toxicity was indicated by a reduction in liver and kidney weight, an increase in SGOT, SGPT, and creatinine levels, and pathological changes in the histoarchitecture of these organs. The decreased levels of CAT, SOD, and GSH, along with a simultaneous increase in lipid peroxidation, suggested oxidative stress. The adverse effects of LCS on spermatogenesis, testosterone levels, oxidative stress, genotoxicity, and cytotoxicity underscore the urgent need for effective waste management and environmental regulation in Deepor Beel.

## CRediT authorship contribution statement

**Ranjit Kakati:** Visualization, Validation, Project administration, Investigation, Data curation, Conceptualization. **Kamal Adhikari:** Writing – review & editing, Writing – original draft, Validation. **Queen Saikia:** Writing – review & editing, Visualization, Software, Investigation, Data curation, Conceptualization. **Ajit Hazarika:** Visualization, Validation, Supervision, Resources.

## Data and code availability

No data was used for the research described in the article.

## Declaration of competing interest

The authors declare that they have no known competing financial interests or personal relationships that could have appeared to influence the work reported in this paper.
